# Editorial

**Published:** 1872-06-15

**Authors:** 


### Medical Examiner.

*A Semi-Monthly Journal of Medical Sciences.*

EDITED BY

N. S. DAVIS, M. D., AND F. H. DAVIS, M. D.

Chicago, June 15th, 1872.

### EDITORIAL.

Explanation.—During the last few months, as most of our readers know, the junior editor has been absent from home, and the amount of work left on the hands of the senior has been such that he has found it impossible to bring the issues of the *Examiner* up to their proper date. Before this paragraph reaches its readers, the junior editor is expected to be again at home and at his post. And we think reliance can be placed in the future prompt and regular issue of each number.

Our Medical Colleges.—The summer course of instruction in the Chicago Medical College, Medical Department of the N.-W. University, closed on the first of July. The course was a pleasant and profitable one, and attended by a good class of students. The college, in all of its departments, is in excellent order, with the ample hospital and dispensary in connection with it, and all are in readiness for the commencement of the next annual course of instruction.

Our friends of the Rush Medical College appear to have abandoned the plan of rebuilding on their old place in the north division of the city, and are constructing a very plain building on the County Hospital lot, on the corner of Eighteenth Street and Arnold. It contains a lecture room and dissecting room, and with the amphitheatre of the hospital, will constitute their accommodations for the coming fall and winter. There is no city in the country that affords better facilities for medical instruction than this. Our schools are well organized; our hospitals are ample, and the arrangements in every department, didactic, demonstrative, and clinical, are complete.

Medical Books.—Those wanting medical books will find a good assortment at the book-store of Messrs. Jansen, McClurg & Co., at 607 Wabash Avenue.

